# Supplementary material for: Uneven distribution of enamel in the tooth crown of a Plains Zebra (Equus quagga)
Source: PeerJ. 2015 Jun 11;3:e1002. doi: 10.7717/peerj.1002 (PMC4465953; doi:10.7717/peerj.1002)
Supplement: Appendix S1 — Voxel sizes of individually scanned teeth. [file peerj-03-1002-s001.docx]

Appendix.1. Voxel sizes of individually scanned teeth.

|  |  |
| --- | --- |
| **Tooth position** | **Voxel size [mm]** |
| P2 | 0.0749 |
| P3 | 0.0855 |
| P4 | 0.0995 |
| M1 | 0.0890 |
| M2 | 0.0934 |
| M3 | 0.0829 |
