# Supplementary material for: Uneven distribution of enamel in the tooth crown of a Plains Zebra (Equus quagga)
Source: PeerJ. 2015 Jun 11;3:e1002. doi: 10.7717/peerj.1002 (PMC4465953; doi:10.7717/peerj.1002)
Supplement: Appendix S2 — Raw data given as voxel counts. When calculated from the individual sections, the voxel count for the whole tooth crown deviates slightly from the number of voxels actually segmented as total volume. In all cases this segmentation error is <1%. [file peerj-03-1002-s002.docx]

|  | **P2** | | | **P3** | | | **P4** | | |
| --- | --- | --- | --- | --- | --- | --- | --- | --- | --- |
|  | total volume | total without cementum | enamel volume | total volume | total without cementum | enamel volume | total volume | total without cementum | enamel volume |
| section 1 | 16084052 | 11702383 | 4397214 | 12010484 | 7897550 | 2782343 | 8716410 | 5935333 | 2373177 |
| section 2 | 11563272 | 9108867 | 3427750 | 9277951 | 6657111 | 2595382 | 5984108 | 4522858 | 1740099 |
| section 3 | 11251917 | 9267336 | 3882362 | 7571577 | 5643475 | 2635101 | 4761440 | 3743875 | 1579430 |
| section 4 | 6687348 | 5906388 | 2782682 | 4531029 | 3513027 | 1650599 | 3330703 | 2536998 | 1386339 |
| whole tooth crown | 45403498 | 35814163 | 14387689 | 33526083 | 23859097 | 9637660 | 22745485 | 16714887 | 7126778 |
| segmentation error | 0.40% | 0.47% | 0.71% | 0.40% | 0.62% | 0.27% | 0.21% | 0.14% | 0.67% |
|  | **M1** | | | **M2** | | | **M3** | | |
|  | total volume | total without cementum | enamel volume | total volume | total without cementum | enamel volume | total volume | total without cementum | enamel volume |
| section 1 | 10405545 | 7082178 | 3419436 | 8535349 | 5363596 | 2494121 | 7548017 | 5345013 | 2865243 |
| section 2 | 11103773 | 8539947 | 3895881 | 8852825 | 6379831 | 2846548 | 5756332 | 4282920 | 2257787 |
| section 3 | 7893412 | 6263552 | 3034753 | 6231951 | 4679182 | 2213797 | 4557893 | 3454044 | 1969033 |
| section 4 | 8186506 | 7328856 | 3466978 | 7472345 | 5992379 | 3030034 | 8552920 | 6934636 | 3794978 |
| whole tooth crown | 37416229 | 28997124 | 13812086 | 31187385 | 22492154 | 10602375 | 26324330 | 19921687 | 10882173 |
| segmentation error | 0.46% | 0.74% | 0.04% | 0.31% | 0.34% | 0.17% | 0.34% | 0.47% | 0.04% |

Appendix 2. Raw data given as voxel counts. When calculated from the individual sections, the voxel count for the whole tooth crown deviates slightly from the number of voxels actually segmented as total volume. In all cases this segmentation error is <1%.
